# Thiophene Derivatives with Antileishmanial Activity Isolated from Aerial Parts of *Porophyllum ruderale* (Jacq.) Cass.

**DOI:** 10.3390/molecules16053469

**Published:** 2011-04-26

**Authors:** Helena Teru Takahashi, Cláudio Roberto Novello, Tânia Ueda-Nakamura, Benedito Prado Dias Filho, João Carlos Palazzo de Mello, Celso Vataru Nakamura

**Affiliations:** 1 Programa de Pós-graduação em Ciências Farmacêuticas, Universidade Estadual de Maringá, Av. Colombo, 5790, 87020-900, Maringá, PR, Brazil; E-Mails: helenatakahashi@yahoo.com.br (H.T.T.); mello@uem.br (J.C.P.M.); 2 Departamento de Farmácia, Universidade Estadual de Maringá, Maringá, PR, Brasil; E-Mail: crnovello@uem.br; 3 Departamento de Ciências Básicas da Saúde, Universidade Estadual de Maringá, Maringá, PR, Brazil; E-Mails: tunakamura@uem.br (T.U.N.); bpdfilho@uem.br (B.P.D.F.)

**Keywords:** *Porophyllum ruderale*, antileishmanial activity, thiophene derivatives, *Leishmania amazonensis*

## Abstract

*Porophyllum ruderale* (Jacq.) Cass. is a plant native to Brazil and in the northwest region of the state of Paraná, Brazil, aerial parts of *P. ruderale* have been used popularly in the treatment of lesions caused by *Leishmania* sp.. In this study the antileishmanial and cytotoxic activities of the crude extract, fractions, and isolated compounds from aerial parts of *P. ruderale* was evaluated. The dichloromethane extract was submitted to chromatography to yield compounds active against *Leishmania amazonensis*. Their structures were established by comparison of their spectroscopic data with literature values. The activities of crude extract against promastigote and axenic amastigote forms of *L. amazonensis* (IC_50_) were 60.3 and 77.7 μg/mL, respectively. Its cytotoxic activity against macrophage cells (CC_50_) was 500 μg/mL. The thiophene derivatives isolated were: 5-methyl-2,2′:5′,2″-terthiophene (compound **A**) and 5′-methyl–[5–(4–acetoxy-1–butynyl)]–2,2′-bithiophene (compound **B**). The activity of compound **A** against promastigote and axenic amastigote forms were 7.7 and 19.0 μg/mL and of compound **B** were 21.3 and 28.7 μg/mL, respectively. The activity of the isolated compounds against promastigote and axenic amastigote forms was better than that of the crude extract and more selective against protozoa than for macrophage cells.

## 1. Introduction

Leishmaniasis is an infection caused by protozoa of the genus *Leishmania*, showing several clinical forms: cutaneous (CL), mucocutaneous (MCL), and visceral (VL) leishmaniasis. This parasitic disease is prevalent in 88 countries (72 of them developing countries) and affects more than 12 million people. More than 90% of VL patients are located in India, Sudan, Brazil, and Bangladesh [1]. Leishmaniasis is a major health problem worldwide, and can be fatal when untreated [2,3,4].

The treatment of leishmaniasis is difficult because of the intramacrophagic location of the infective form. Victims of this illness are usually immunodeficient and are not able to eliminate the parasites through a natural mechanism of defense. Moreover, malnutrition is associated with certain cases of leishmaniasis. Concurrent infections such as malaria and pneumonia increase the fatality rate of the illness if it is not promptly diagnosed and treated. The problem of leishmaniasis has been worsened by the spread of Acquired Immune Deficiency Syndrome (AIDS), due to parallel infections in AIDS patients; as well as by the development of drug resistance by the parasites [5,6]. No vaccines are available for any form of the disease, and the chemotherapy is still inadequate and expensive [7], therefore there is an urgent need for new chemotherapeutic drugs for the treatment of these diseases, which mainly affect people in developing countries.

Extensive studies have shown that medicinal plants in several regions of the world contain compounds active against protozoa [8]. *Porophyllum ruderale* (Jacq.) Cass. (*Asteraceae*) is a medium-sized ruderal aromatic herb shrub with a strong fragrance [9]. It is native to Brazil, where it is considered invasive because it adapts to many soil types, including poor and sandy ones, and is common in the southeastern region of the country [10]. It is used in folk medicine against leishmaniasis, for cicatrization, general pain, and internal bruising [11,12]. Some pharmacological activities have been evaluated, including anti-inflammatory [13], insecticidal [14], analgesic and antispasmodic [15], antifungal and antibacterial [16], and photoprotective properties [17]. In the northwest region of the state of Paraná (Brazil), aerial parts from *P. ruderale* have been used popularly in the treatment of lesions caused by *Leishmania* sp. [12]. In the present study, we evaluated the *in vitro* antileishmanial and cytotoxic activities against J774G_8_ macrophage cells of the crude extracts, fractions, and two thiophene derivatives isolated from aerial parts of *P. ruderale*.

## 2. Results and Discussion

Although leishmaniasis has been known for a long time [18], it is still considered a public health problem, mainly in developing countries. Brazil is among the countries with the highest reported incidence rates, with a mean of 28,000 new cases of cutaneous leishmaniasis (CL) and 2,000 new cases of visceral leishmaniasis (VL) per year [19]. A new CL endemic zone was recently reported in city of Prudentópolis in the central part of the state of Paraná in southern Brazil [20].

The available chemotherapy for treatment of leishmaniasis is not very effective, and is highly toxic to patients. Therefore, there is an immediate need for new treatment alternatives [7]. Approximately 80% of the world population uses traditional medicine, primarily based on natural products [21]. According to Rocha *et al.* [22], the potent leishmanicidal activities of chemically defined molecules isolated from natural origins represent an exciting advance in the search for novel antiprotozoal agents, at a time when the need for new innovative drug leads is urgent. In addition, the costs of treating this disease need to be reduced as much as possible, to allow treatments to be disseminated and used mainly in poorer countries, where there is a high incidence of this disease [23].

Previous phytochemical analysis of *P. ruderale* showed the presence of several constituents, including: essential oils [13,16], monoterpenes and sesquiterpenes [14], carotenoids, fatty acids, alkaloids, coumarins, catechin tannins, quaternary amines, flavonoid and aglycone flavones, anthocyanins, polysaccharides, triterpene or steroidal saponins, and mucilage [15]. In the present study, we isolated two compounds by chromatographic separation of a dichloromethane extract of aerial parts from *P. ruderale*., which were identified by their MS and NMR spectra analysis as thiophene derivatives: 5-methyl-2,2′:5′,2″-terthiophene and 5′-methyl–[5–(4–acetoxy-1–butynyl)]–2,2′-bisthiophene. Both compounds were more active than the crude dichloromethane extract or the active fraction obtained from *P. ruderale*. Terthiophene derivatives isolated from plants have shown antifungal [24,25] and antibacterial activity [26]. Terthiophenes and polyenes isolated from species of the family *Asteraceae* have shown activity against microorganisms, viruses, and tumor cells [27].

**Figure 1:**
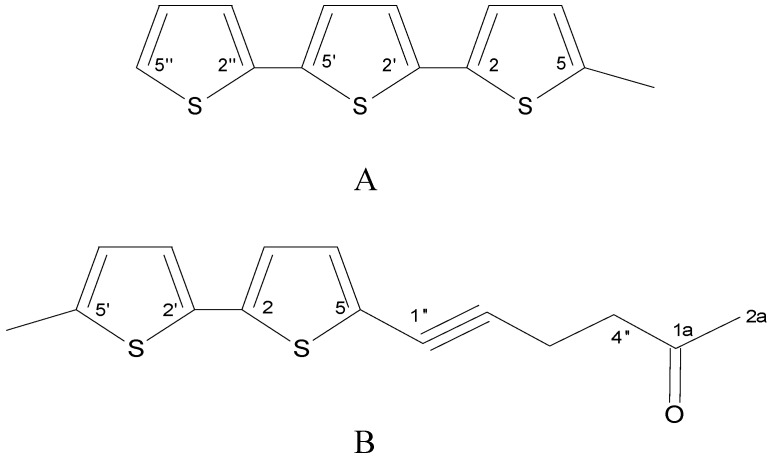
Structures of the thiophene derivatives isolated from *P. ruderale* (**A**) 5-methyl-2,2′:5′,2″-terthiophene; (**B**) 5′-methyl-[5-4(4-acetoxy-1-butynyl)]-2,2′ bi-thiophene.

Marotti *et al.* [28] isolated thiophene and polyacetylenic compounds from species of *Tagetes*. These compounds showed strong biocidal activity, thus making *Tagetes* plants very useful for suppressing soil nematode populations and as sources of natural pesticides.

The effect of the crude extract obtained from the aerial parts of *P. ruderale* was verified on the growth of *L. amazonensis*. The dichloromethane extract inhibited the growth of the promastigote and axenic amastigote forms. The bioguided fractionation led to isolation of thiophene derivative compounds (**A** and **B**) that showed better activity than the crude extract against both forms of the parasite (Table 1).

Jorge *et al.* [12] investigated the activity of crude extract of *P. ruderale* against promastigote forms of *L. braziliensis* and observed an inhibition of 22.7%, 41.0% and 63.6% at 24, 48 and 72 h of incubation with concentrations of 1.5 and 2.5 mg/mL. In the present study, 60.3 µg/mL of dichloromethane extract of *P. ruderale* inhibited 50% of promastigote forms of *L. amazonensis* after 72 h of incubation.

The thiophene derivatives: 5-(4-hydroxy-1-butynyl)-2,2′-bithienyl and 5-(4-acetoxy-1-butynyl)-2,2′-buthienyl from *Tagetes mendocina* (*Asteraceae*) showed antiprotozoal activity against *Leishmania amazonensis, L. brasiliensis* and *L. infantum* promastigotes with 100% lysis at 100 μg/mL [29]. In our study, we found a 50% Inhibitory Concentration (IC_50_) against *L. amazonensis* of 7.7 and 21.3 μg/mL for compounds **A** and **B**, respectively. The IC_50_ values for the positive control, amphotericin B, were relatively low, at 0.060 μg/mL against the promastigotes and 0.23 μg/mL against the amastigotes.

The cytotoxic effects of the dichloromethane extract, active fractions, and two thiophene derivatives were evaluated by the sulforhodamine B method. The 50% cytotoxic concentrations were determined, and the activities against the protozoa were compared using the selectivity index (SI) ratio (CC_50_ J774G_8_ cells/IC_50_ protozoa). Cytotoxicity assays demonstrated that compounds **A** and **B** were 48.2 and 15.7 times more toxic to promastigotes and 19.1 and 11.7 times more toxic to intracellular amastigote forms, respectively, than to the J774G_8_ macrophage cells (Table 1).

In view of the present clinical scenario, it is desirable to develop new drugs that are affordable, less toxic, and more effective. In developing countries, people are almost completely dependent on traditional medical practices for their primary health care, and higher plants remain the main source of drug therapy in traditional medicine. The recognition and validation of traditional medical practices and the search for plant-derived drugs could lead to the development of new strategies for leishmaniasis control and treatment [22,30,31,32]. In recent years, several studies with plants used in folk medicine have been performed to assess their antileishmanial activity [33,34,35,36,37].

In the northwest of Paraná, the aqueous, hydroalcoholic or even the juice of fresh aerial parts of *P. ruderale* has been popularly employed in the treatment of leishmaniasis by applying the same on skin lesions caused by *Leishmania* sp. In our *in vitro* study, we found that the dichloromethane extract of *P. ruderale* as well as the isolated compounds inhibited the growth of promastigotes and amastigotes of *L. amazonensis*. These are likely candidates for selection as lead compounds for the development of new drugs against leishmaniasis; however, their antileishmanial activity, toxicity *in vivo*, and their detailed mechanism of action must all be evaluated with care.

## 3. Experimental

### 3.1. General

The NMR spectra were obtained in a Varian Gemini 300 (7.05T) instrument using CDCl_3_ as the solvent for field homogeneity, TMS as the internal standard, and a constant temperature of 298 K. For HMBC the coupling constants were optimized for 4, 6, 8, and 12 Hz. ESI-MS was recorded in a Quatro LCZ Micromass mass spectrometer (Waters, Manchester, UK).

### 3.2. Plant material

Aerial parts of *P. ruderale* were collected in Cascavel, Paraná, Brazil, in 2009. An exsiccate of the collected and identified plant was deposited and authenticated at the Herbarium of the State University of Maringá, Maringá, Brazil, under number HUEM 10074.

### 3.3. Preparation of dichloromethane extract

Aerial parts (2,900 g) from *P. ruderale* were collected, cut into small pieces with scissors, and extracted by dynamic maceration with 12.5% (v/w) dichloromethane (Synth^®^) at room temperature in the dark for 2 days, resulting in a greenish solution. This extract was filtered on filter paper (Whatman 40^®^), concentrated under reduced pressure at a temperature lower than 40 °C, and lyophilized to give 22.96 g of dried extract (0.79%). This dichloromethane extract was submitted to VLC.

### 3.4. Isolation and identification of active compounds

The dichloromethane extract (1.5 g) was added to a silica-gel 60 (0.063–0.200 mm, Merck^®^) Vacuum Liquid Chromatography (VLC) column (40 cm × 1.8 cm) and eluted successively with petroleum ether, petroleum ether-diethyl ether (15:1; 10:1; 7:1; 1:1 v/v), diethyl ether, diethyl ether-methanol (1:1), ethyl acetate-methanol (8:2; 1:1 v/v), and methanol. The fractions were monitored by thin-layer chromatography (TLC) using silica gel F_254_ plates (Merck) and the following eluent systems: hexane-petroleum ether (5:1 v/v); and petroleum ether-diethyl ether (2:1; 2:1.5; 1:1 v/v). Similar fractions were combined, yielding 18 fractions (F_1_ to F_18_). The F_3_ fraction (12.8 mg, 0.007%) was identified as 5-methyl-2,2′:5′,2″-terthiophene by NMR and mass spectroscopy (MS) analysis. The active fraction F_13_ (175.0 mg) was separated by column chromatography (CC, 64 × 1.0 cm) with silica-gel 60 (0.063–0.200 mm, Merck) and eluted with petroleum ether-diethyl ether (9:1 v/v). Fractions were checked by TLC using hexane-petroleum ether (5:1 v/v); and petroleum ether-diethyl ether (1:0.2, 1:0.5 v/v) as eluents and similar ones were combined to obtain 16 subfractions (F_13.1_ to F_13.16_). Subfraction F_13.7_ (35.5 mg, 0.018%) was identified by MS and NMR analysis as 5′-methyl–[5–(4-acetoxy-1-butynyl)]-2,2′ bisthiophene– compound **B**.

*5-Methyl-2,2′:5′,2″-terthiophene* (**A**) [38]. Viscous yellow oil. ^1^H-NMR δ: 2.47 (3H, bs, 5-Me), 6.65 (1H, dd, *J* = 3.6, 1.2 Hz, H-4), 6.95 (1H, d, *J* = 3.6 Hz, H-3′), 6.97 (1H, d, *J* = 3.6 Hz, H-3), 7.00 (1H, dd, *J* = 5.1, 3.6 Hz, H-3″), 7.04 (1H, d, *J* = 3.6 Hz, H-4′), 7.14 (1H, dd, *J* = 3.6, 0.9 Hz, H-4″), 7.19 (1H, dd, *J* = 5.1, 0.9 Hz, H-5″). ^13^C-NMR δ: 15.6 (5-Me), 123.6 (C-4″), 123.7 (C-3′), 123.8 (C-4′), 124.4 (C-3″), 124.5 (C-5″), 126.2 (C-4), 128.0 (C-3), 135.0 (C-2), 135.7 (C-5′), 136.9 (C-2′), 139.6 (C-5), 137.5 (C-2″). ESI-MS *m/z* (Rel. Int. %): 185 (15), 229 (25), 263 [M+H]^+^ (36), 262 (100). Molecular formula: C_13_H_10_S_3_.

*5′-Methyl-[5-(4-acetoxy-1-butynyl)]-2,2′-bi-thiophene* (**B**) [39]. Yellow oil. ^1^H-NMR δ: 2.10 (3H, s, H-2a), 2.47 (3H, d, *J* = 0.9 Hz, 5′-Me), 2.78 (2H, t, *J* = 6.9 Hz, H-3″), 4.26 (2H, t, *J* = 6.9 Hz, H-4″), 6.65 (1H, dd, *J* = 3.6, 1.2 Hz, H-4′), 6,90 (1H, d, *J* = 3.6 Hz, H-3), 6.95 (1H, d, *J* = 3.6 Hz, H-3′), 7.01 (1H, d, *J* = 3.9 Hz, H-4). ^13^C-NMR δ: 15.57 (5′-Me), 20.5 (C-3″), 21.1 (C-2a), 62.3 (C-4″), 77.6 (C-1″), 90.6 (C-2″), 121.6 (C-5), 122.7 (C-3), 124.2 (C-3′), 126.3 (C-4′), 132.6 (C-4), 140 (C-5′). ESI-MS *m/z* (Rel. Int. %): 291 [M+H]^+^ (10), 301 (26), 245 (33), 229 (73), 313 [M+Na]^+^ (100). Molecular formula: C_15_H_14_S_2_O_2_.

### 3.5. Antileishmanial activity

#### 3.5.1. Antipromastigote activity

*L. amazonensis* promastigotes (strain WHOM/BR/75/JOSEFA) were grown at 25 °C in Warren’s medium supplemented with 10% (v/v) heat-inactivated fetal bovine serum (FBS), for 48 h. The cells were harvested, resuspended in fresh medium, counted in a Neubauer chamber, and adjusted to a concentration of 1 × 10^6^ cells/mL. To evaluate biological activities, the following concentrations were used: from 10 to 1,000 μg/mL for crude extracts, 10 to 500 μg/mL for fractions, and 1 to 100 μg/mL for isolated compounds; all were aseptically solubilized in Dimethylsulfoxide (DMSO) - the highest concentration used was 1.0% v/v and incubated at 25 °C. After 72 h of incubation, the parasites were counted in a Neubauer chamber. All tests were done in duplicate. The 50% inhibitory concentration (IC_50_) represents the concentration that causes 50% inhibition in parasite growth.

#### 3.5.2. Anti-amastigote activity

Axenic amastigotes were cultured in Schneider’s Insect Medium (Sigma Chemical Co., St. Louis, MO, U.S.A.), supplemented with 20% inactivated FBS containing dichloromethane extract or thiophene derivatives in 12-well microplates at 32 °C, for 72 h. The cell density for each treatment was determined in a hemocytometer (Improved Double Neubauer) with an optical microscope. In all tests, 1% DMSO (Sigma Chemical Co), at the concentration applied to dissolve the highest dose of the compounds that had no effect on cell proliferation, and medium alone were used as controls. Each experiment was performed in triplicate on three different occasions.

### 3.6. Cytotoxicity and selectivity index

The cytotoxicity test was performed according to Skehan *et al.* [40]. A suspension of 5 × 10^4^ J774G_8_ macrophage cells in RPMI 1640 medium supplemented with 10% FBS was added to each well in a 96-well microplate. The plates were incubated in a 5% CO_2_–air mixture at 37 °C to obtain confluent growth of the cells. After 24 h, the medium was removed, and one of several concentrations of the crude extract (100 to 5,000 μg/mL), active fraction, or isolated compounds (10 to 1,000 μg/mL) was added to each well containing the cells, and the plates were incubated for 48 h. The nonadherent cells were removed by washing with RPMI 1640 medium, and the adhered macrophages were fixed with 50 μL/well of 10% trichloroacetic acid at 4 °C for 1 h; after that, they were washed with water, and 50 μL/well of sulforhodamine B (0.4% w/v) was added; the microplate was then maintained at 4 °C for 30 min. Next, the sulforhodamine B was removed, and the microplate was washed five times with 1% acetic acid, then 150 μL/well of 10 mM unbuffered Trisbase solution (Sigma Chemical Co.) was added, and it was homogenized for 15 min. Next, the absorbance of each individual well was read in a 96-well plate reader (BIOTEK Power Wave XS) at 530 nm. Each experiment was performed in triplicate on three different occasions. The percentage of viable cells was calculated in relation to the control cultured in the medium alone. The 50% cytotoxicity concentration (CC_50_) was determined by logarithm regression analysis of the data obtained.

## 4. Conclusions

Our results showed that the dichloromethane extract, active fraction, and thiophene derivatives isolated from *P. ruderale* exhibit strong activity against promastigote and axenic amastigote forms of *L. amazonensis*.

## Figures and Tables

**Table 1:** Effects of the dichloromethane extract, active fraction and thiophene derivatives on the growth of promastigote (PRO) and axenic amastigote forms (AMA) of *L. amazonensis* and cytotoxicity to J774G_8_ Macrophage Cells.

	IC_50_ (µg/mL)		CC_50_ (µg/mL)	SI	
	PRO	AMA	Macrophages	PRO	AMA
Dichloromethane extract	60.3 ± 9.2	77.7 ± 7.7	500 ± 50	8.3	6.5
Active fraction	57.5 ± 4.5	72.5 ± 7.5	440 ± 56.6	7.6	7.8
Compound **A**	7.7 ± 1.7	19.0 ± 4.7	370 ± 50	48.2	19.1
Compound **B**	21.3 ± 4.4	28.7 ± 2.6	335 ± 15	15.7	11.7
Amphotericin B	0.06 ± 0.0	0.23 ± 0.0	ND	ND	ND
